# A Randomized Controlled Trial of Attentional Control Training for Treating Alcohol Use Disorder

**DOI:** 10.3389/fpsyt.2021.748848

**Published:** 2021-11-26

**Authors:** Angelina Isabella Mellentin, W. Miles Cox, Javad S. Fadardi, Laila Martinussen, Nicolaj Mistarz, Lotte Skøt, Kristine Rømer Thomsen, Kim Mathiasen, Mia Lichtenstein, Anette Søgaard Nielsen

**Affiliations:** ^1^Unit for Psychiatric Research, Department of Clinical Research, University of Southern Denmark, Odense, Denmark; ^2^Brain Research-Inter-Disciplinary Guided Excellence (BRIDGE), Department of Clinical Research, University of Southern Denmark, Odense, Denmark; ^3^Centre for Telepsychiatry, Mental Health Services in the Region of Southern Denmark, Odense, Denmark; ^4^School of Human and Behavioral Sciences, Bangor University, Bangor, United Kingdom; ^5^Cognitive Health Laboratory, Department of Psychology, Ferdowsi University of Mashhad, Mashhad, Iran; ^6^Centre for Alcohol and Drug Research, Department of Psychology and Behavioral Sciences, Aarhus University, Aarhus, Denmark

**Keywords:** alcohol use disorder, attentional bias, Stroop task, cognitive bias modification, add-on treatment, randomized clinical trial

## Abstract

**Background:** There is consistent evidence that community and clinical samples of individuals with an alcohol use disorder (AUD) have attentional biases toward alcohol cues. The alcohol attentional control training program (AACTP) has shown promise for retraining these biases and decreasing alcohol consumption in community samples of excessive drinkers. However, there is a lack of evidence regarding the effectiveness of ACTP in clinical AUD samples. The main aim of the present study is to investigate whether primary pharmacological and psychological, evidence-based alcohol treatment can be enhanced by the addition of a gamified AACTP smartphone application for patients with an AUD.

**Design and Methods:** The study will be implemented as a randomized controlled trial. A total of 317 consecutively enrolled patients with AUD will be recruited from alcohol outpatient clinics in Denmark. Patients will be randomized to one of three groups upon initiation of primary alcohol treatment: Group A: a gamified AACTP smartphone application + treatment as usual (TAU); Group B: a gamified AACTP sham-control application + TAU; or Group C: only TAU. Treatment outcomes will be assessed at baseline, post-treatment, and at 3- and 6-month follow-ups. Repeated measures MANOVA will be used to compare the trajectories of the groups over time on alcohol attentional bias, alcohol craving, and drinking reductions. It is hypothesized that Group A will achieve better treatment outcomes than either Group B or Group C.

**Perspectives:** Because attentional bias for alcohol cues is proportional to the amount of alcohol consumed, and these biases are not addressed within current evidence-based treatment programs, this study is expected to provide new evidence regarding the effectiveness of the gamified AACTP in a clinical population. Furthermore, due to promising results found using AACTP in community samples of excessive drinkers, there is a high probability that the AACTP treatment in this study will also be effective, thereby allowing AACTP to be readily implemented in clinical settings. Finally, we expect that this study will increase the effectiveness of evidence-based AUD treatment and introduce a new, low-cost gamified treatment targeting patients with an AUD. Overall, this study is likely to have an impact at the scientific, clinical, and societal levels.

**Clinical Trial Registration:** https://clinicaltrials.gov/ct2/show/NCT05102942?term=NCT05102942&draw=2&rank=1, identifier: NCT05102942.

## Background

Alcohol-use disorder (AUD), as defined by DSM-5 criteria, is very common in the Western world, with a 12-month prevalence of 14% and a lifetime prevalence of 29% (1). Furthermore, AUD is the most prevalent and most harmful of all substance-use disorders (2, 3) and is among the leading causes of illness, disability, and mortality (4, 5). The high prevalence and severity of AUD underscore the importance of having easily available and effective treatments.

Overall, evidence-based psychological treatments, such as cognitive-behavioral therapy (CBT), have shown to be effective in the short term (6). During CBT, patients with an AUD are taught methods to help them identify high-risk situations for relapse. They also learn coping strategies for avoiding alcohol in high-risk situations. Patients are, however, supposed to learn these strategies by applying explicit and controlled cognitive techniques rather than practicing the strategies while being exposed to alcohol *in vivo* (7–9). Recovering patients often experience a relapse when they are exposed to alcohol and related stimuli in real-life, and they can only later analyze the consequences of their behavior. Patients with an AUD initially respond well to evidence-based psychological treatments, but as many as 50% of them relapse within 6 months after treatment discharge (10–12). This indicates that CBT and other evidence-based psychological treatments do not address all the crucial cognitive dysfunctions associated with maintaining an addiction; thus, they may not prepare patients to deal with their inevitable confrontation with alcohol cues in real life in Western societies.

One shortcoming of current psychological treatments is that they mainly target explicit and controlled cognitive dysfunctions. However, according to dual process models of addictive behaviors, confrontation with alcohol cues *in vivo* is influenced by two semi-independent cognitive systems: (1) a fast associative *impulsive* system, which is involved in the automatic evaluation of alcohol-related stimuli in the environment in terms of their emotional and motivational significance and which initiates approach or avoidance responses; and (2) a slow and controlled *reflective* system, which is involved in the regulation of the automatic and implicit responses elicited by the impulsive system, and which is responsible for explicit and controlled, higher-order cognitive processes (13–16).

Addictive behaviors, such as AUD, can be conceptualized as a dysfunction of these systems, whereby an over-activated impulsive system becomes sensitized and triggers approach behaviors toward alcohol cues, whereas a relatively under-activated reflective system is unable to regulate the behavior. Because the impulsive system is partly automatic and implicit, approach behaviors targeting alcohol cues in the environment allow the self-destructive drinking behavior to be maintained, even though the drinker might have explicit knowledge about the consequences generated by the reflective system (15, 16). Corroborating evidence from functional neuroimaging studies suggest that patients with AUD exhibit aberrant activation in dorsolateral prefrontal cortex, insular cortex, and anterior cingulate cortex, which are structures that act as a critical part of the reflective system (17–21). In addition to the of hypoactivation of the neural correlates for the reflective system, hyperactivation of subcortical structures such as ventral striatum and nucleus accumbens have also been found in AUD, which underlines the notion of a dysfunctional interaction between the reflective and impulsive systems (19–22). Although standard evidence-based psychological treatments for AUD target and attempt to modify dysfunctions in one of the cognitive systems that influence the maintenance of an AUD, the impulsive cognitive dysfunctions are addressed to a much lesser extent.

A cognitive dysfunction in the impulsive system that has consistently been identified as a crucial component in the maintenance of an AUD is alcohol attentional bias (AB). An AB for alcohol cues refers to the implicit and automatic cognitive process of selectively focusing attention on alcohol-related stimuli in the environment at the expense of processing other relevant stimuli (23, 24). AB for alcohol cues has been measured by using a variety of experimental paradigms (25). Among the most widely used paradigm is the alcohol variant of the experimental Stroop task (24, 26, 27). The alcohol Stroop consists of two categories of stimuli: alcohol-related (e.g., beer, wine, tavern) and neutral (e.g., chair, envelop, juice). During the task participants are asked to name the color (i.e., red, yellow, blue, or green) in which each stimulus appears as fast and accurately as possible while ignoring their content and meaning. The degree of AB is indicated by slower mean reaction times to alcohol cues compared to neutral cues and is calculated as an interference score. The alcohol-related meaning of the stimuli is presumably either capturing the person's attention more readily or maintaining the attentional focus for a longer period of time than the neutral cues (24). AB as measured by the alcohol Stroop task has consistently been found in both community and clinical samples of excessive drinkers (28–31), and the degree of AB is proportional to the amount of alcohol that participants habitually consume (24). For example, clinical AUD samples exhibit greater AB than sub-clinical samples of heavy drinkers (24, 29, 32). In addition, the degree of AB is inversely related to drinkers' ability to control their drinking (31, 33–35). Finally, it has been shown that clinical samples of AUD patients who have stronger alcohol AB at treatment admission are more likely to have an unsuccessful treatment outcome compared to patients with less AB, and the degree of AB of the unsuccessful patients increased during the course of treatment, suggesting that abstinence increases attentional awareness of alcohol cues (28). Taken together, the research suggests that AB is of utmost importance in maintaining an AUD and if not corrected, it might impede the effectiveness of other treatments and cause the patient to relapse.

**Figure 1:**
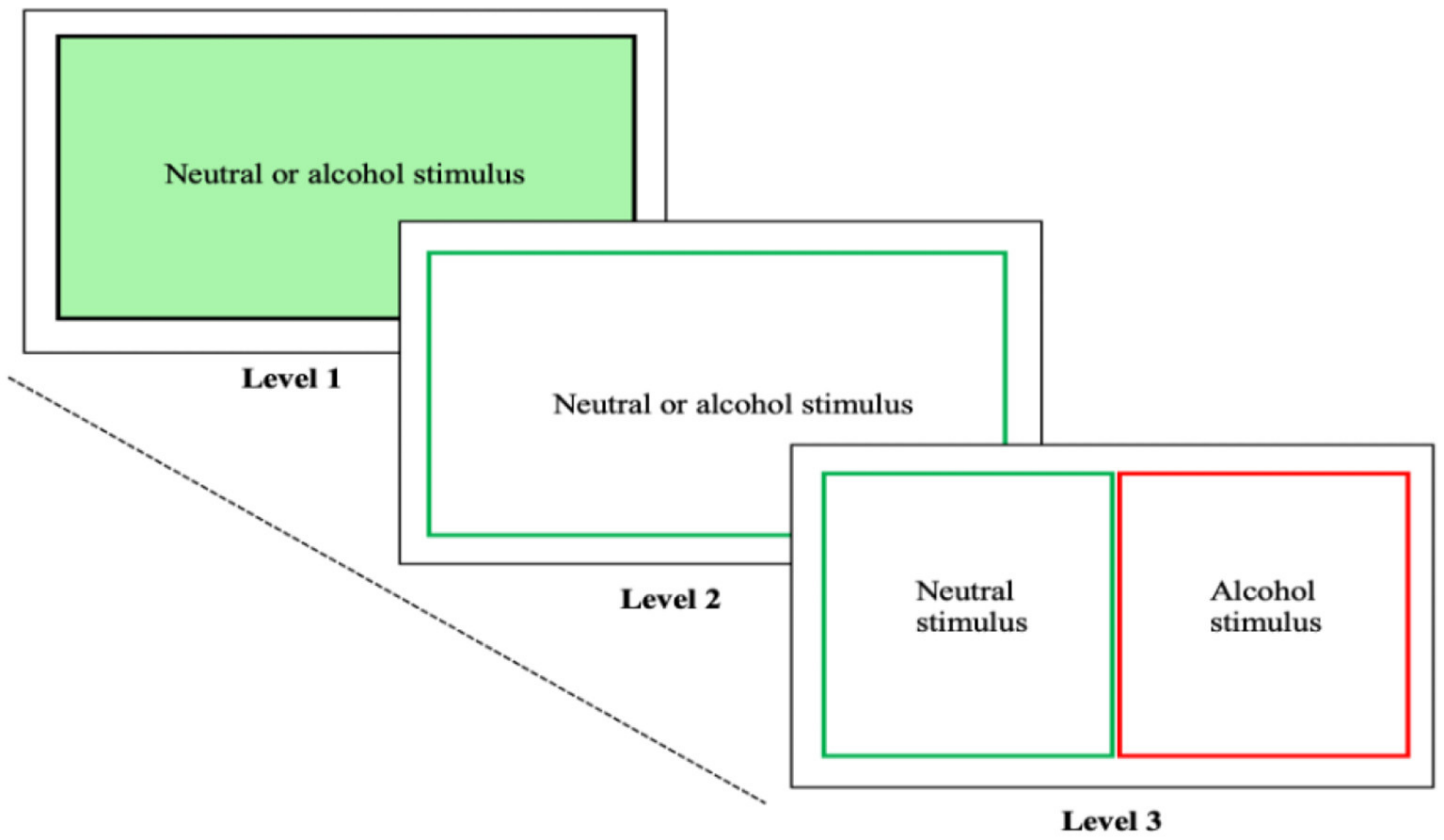
Alcohol attention control training program: In Level 1, neutral or alcohol stimuli are successively presented on the screen, while the colored background of the screen needs to be identified. In Level 2, instead of the background, a colored outline needs to be identified. In Level 3, neutral and alcohol stimuli appear simultaneously of which the neutral stimulus needs to be identified.

The principles underlying the experimental alcohol Stroop task have recently been applied in the Alcohol Attention Control Training Program (AACTP), which is a computerized training program aimed at increasing drinkers' cognitive control over their alcohol AB. As illustrated in Figure 1, the AACTP consists of three different levels of training.

At Level 1, individual pictorial stimuli with a colored background (e.g., red, or green) are shown one at a time on a computer screen. Each stimulus is either alcohol-related or neutral. The participant is instructed to ignore the content of the stimuli while naming the color of the background. At Level 2, again an alcohol-related and a neutral stimulus is presented successively on the computer screen one at a time. In contrast to Level 1 in which the pictures have a colored background, the pictures now have only a colored outline whose color the participant is supposed to identify, thus making the task more difficult than at Level 1. At Level 3, two stimuli (an alcohol-related one and a neutral one) are presented simultaneously on the computer screen. The participant is asked to identify as quickly and accurately as possible the outline color of the neutral stimulus, while ignoring the alcohol-related stimulus.

The AACTP uses a variety of pictorial alcohol-related stimuli to train participants to progressively divert their attention away from alcohol-stimuli, with increasing levels of difficulty across the training. The alcohol AACTP has been evaluated in community samples of excessive drinkers who have shown reductions in both AB and alcohol consumption at post-treatment and follow-up (32, 36, 37). Recent reviews and meta-analyses do point to methodological shortcomings and conflicting evidence on the effects of training programs targeting AB across patients with various addictive disorders, including AUD (38–40). Despite the need for more well-designed studies, there is, however, also evidence suggesting that AACTP may be more promising for targeting alcohol consumption when compared to other AB training programs (40). Furthermore, the ACTP has been studied as an add-on treatment among clinical populations with food- and opiate addictions. In both cases, the addition of the ACTP in the experimental group led to a greater reduction in disease-specific AB and the accompanying unhealthy behavior than in the control groups (41, 42). This evidence suggests that AACTP might be a promising tool for ameliorating AB in clinical samples of patients with an AUD, especially when used in conjunction with a conventional treatment for AUDs.

In contrast to many other psychological treatments, clinical neuropsychological treatments building on experimental laboratory tasks are particularly suitable for alternative delivery pathways, such as tablets and mobile applications. These delivery pathways make it possible to use gamification elements, which would increase patients' motivation to participate. Gamification is defined as the use of game-design features in a non-gaming context, such as in a psychological treatment setting. Gamification would make the intervention more engaging, enjoyable, and motivating for participants (43), and using gamification elements is likely to enhance compliance with neuropsychological treatments. Commonly used gaming techniques include time-pressure, sound effects, feedback, progression in level of difficulty, achievement rankings, and competition (43–45). Although the AACTP have shown great promise in treating cognitive dysfunctions not targeted by conventional psychological treatments, the use of gamification elements may enhance compliance with and the effectiveness of these interventions.

Taken together, the results of prior studies are promising in terms of the clinical relevance of AB training. However, the small number of studies with small sample sizes conducted to date highlights the need for future research to verify the robustness of these mostly positive findings in actual clinical AUD settings (40). Further, adding gamification elements may increase compliance and hence the effectiveness of the intervention.

### Aims

The main aim of the study is to determine whether a gamified version of the AACTP when used as an add-on to a primary evidence-based treatment will increase the treatment's effectiveness. Based on the available evidence, it is hypothesized that the experimental group (Group A) will achieve a better treatment outcome than either the active control group (Group B) or the treatment-as-usual group (Group C). Additionally, we hypothesize that there will be a dose-response effect in Group A: the more that the patients use the AACTP, the better their outcome will be. Full descriptions of the experimental and control groups are provided in Section 2.5 below.

## Methods

The study (a) will be registered in ClinicalTrials.gov, which is the largest registry of clinical trials and is administered by the United States National Library of Medicine (NLM) at the National Institutes of Health, and (b) will be conducted and reported according to CONSORT guidelines (46).

### Design and Setting

The study will be conducted as a parallel randomized controlled study in various outpatient alcohol treatment clinics in Denmark. Most individuals with AUD who present themselves for treatment at these clinics are offered outpatient treatment. This outpatient treatment is financed through public funds. It accepts self-referrals, and patients can remain anonymous during treatment. Only treatment for alcohol problems is provided at the outpatient clinics.

### Primary Treatment at the Outpatient Alcohol Clinics

At treatment entry, patients will be offered detoxification, if needed, before their primary treatment at one of the outpatient clinics is initiated. The primary treatment lasts 3 months and consists of both a pharmacological and a psychological treatment. The pharmacological treatment includes treatment with disulfiram and acamprosate. Naltrexone or nalmefene is also offered when deemed appropriate. The psychological treatment consists of cognitive-behavioral therapy (CBT), which is provided during hour-long individual or group sessions and typically includes eight sessions. The therapist and patient jointly plan the course of the patient's treatment. The treatment typically includes psychoeducation, functional analysis of drinking situations, and the development of coping strategies. The strategies include waiting out until an urge passes rather than acting on it, thinking about the negative consequences of drinking, thinking about the positive consequences of sobriety, consuming healthy alternative foods and beverages, problem-solving, and homework assignments between sessions. Therapists conduct the psychological treatment; they include nurses and social workers who have been specifically trained to deliver the range of therapies. Supervision takes place frequently, and psychiatrists regularly monitor patients' progress during the treatment (47).

### Recruitment

Based on a power calculation (see Section 2.7.), a total of 317 patients will be required for the data analysis. On the basis of data from the Danish National Alcohol Treatment Register (48) and prior studies conducted at this clinic (49–52) and prior studies of AACTP (32, 36, 42), it seems feasible that at least 317 eligible patients (see Section 2.4.) can be recruited from January 2021 to July 2022.

After completing detoxification and prior to starting primary treatment, each patient will be briefly informed about the project and asked whether he or she is willing to meet with a research assistant who will provide further information about the study. If the patient agrees, the research assistant will provide the patient with oral and written information about the details of the study. After informed consent has been provided, a baseline interview will be conducted, and each patient meeting the eligibility criteria will be randomized. To minimize the risk of bias and to overcome potential group imbalances, the current study will utilize an urn technique that will randomize the patients to one of the three treatment groups.

### Eligibility Criteria

To be eligible to participate, patients must fulfill the following criteria. They must (1) sign written informed consent, (2) be between 18 and 65 years old (because the intervention is web-based), (3) be fluent in Danish, (4) have completed detoxification (if deemed appropriate), (5) have been admitted to primary treatment within the past 8 weeks, (6) not be color-blind, (7) not have a severe psychiatric or neurological illness (e.g., a psychotic disorder, intellectual disability, dementia) or terminal physical illness.

### Experimental and Control Groups

The 317 patients (see power analysis below) fulfilling the eligibility criteria will be randomized to one of the three groups: Group A: AACTP delivered *via* a smartphone application + treatment as usual (TAU; *n* = 106), Group B: ACTP sham training delivered *via* a smartphone application + TAU (*n* = 106), or Group C: TAU only (*n* = 106). Patients in Group A will receive seven sessions of AACTP (one session per week for 7 weeks). Patients in Group B will receive seven sessions of sham training (one session per week for 7 weeks). Patients in Group C will receive only the primary treatment for AUD. Patients in the AACTP and sham control groups will start the primary treatment within the 1st month of their admission, so that the add-on treatment will not extend beyond the 3-month treatment period.

#### Attentional Control Training Program *via* a Smartphone Application

The AACTP, which is delivered *via* a smartphone application and based on an Android application, was designed in accordance with the conventional AACTP to help drinkers become aware of the automatic cognitive aspects of their alcohol use. The stimuli comprise alcohol-related pictures and non-alcohol-related pictures and are individually presented on a computer screen in a random order. If patients need it, a research assistant in the treatment facility will assist them with the installation of the application on their smartphone. The installation of the application on patients' personal smartphone will allow them to complete the training program at home. Thus, they will not be constrained by having to use the application in the outpatient facility.

The AATCP training will consist of seven sessions, each of which takes 10–15 min to complete. Each session will start with practice with individual stimuli (i.e., Levels 1 and 2 in the original AACTP) and proceed to Level 3, in which the patient will be asked to either (a) identify the green outline color of a non-alcohol-related picture, while ignoring the red outline color of the alcohol-related picture presented adjacent to it (stimulus-irrelevant version), or (b) identify the picture with non-alcohol-related content, while ignoring the picture with alcohol-related content presented adjacent to it (stimulus-relevant version). Level 3 is the most difficult and most effective level in the conventional AACTP. It is defined as the level in which the patient is trained to divert his or her attention away from alcohol cues when both an alcoholic and a non-alcoholic stimulus is present, and they compete for the patient's attentional resources. The patient should respond by touching the non-alcohol stimulus on the screen as fast and accurately as possible. During the sessions, the speed at which the stimuli are presented systematically increases, while the width of the outlines decreases and gradually fades away in the final sessions. This increases the difficulty of the task and trains the person's attentional system so that the alcohol-related stimuli are ignored even when there is no color cue. In case the patient makes a mistake and touches the incorrect stimulus, the tablet will provide an auditory-visual alert.

The training, therefore, occurs in seven hierarchical steps, which are arranged according to an increasing level of difficulty. Prior to each session, the patient will be encouraged to set a goal for decreasing his or her reaction times (RTs) to the neutral, non-alcohol-related stimuli, which requires increasing efficiency for the distracting cues to be ignored. The goal will be for each participant to improve his or her attentional control until a performance plateau has been reached (RTs < 1,000 ms with a response accuracy > 90%).

After completing each session, the participant will be given numerical/graphical feedback on (a) the number of errors made and the mean RTs to the non-alcohol-related stimuli, and (b) a brief auditory/written interpretation of the results. The aim of the training will be to motivate patients to actively take part in the program in a meaningful and goal-directed way. Although the conventional AACTP includes some gamification elements, the smartphone application includes additional gamification elements, such as time-pressure, sound-effects, hierarchical levels, feedback, achievement ranking, and competition. These elements serve to increase the patient's engagement and compliance. Also, to increase engagement and the chances of obtaining a dose-response effect across participants, the application can control for the number of practice sessions that each trainee can complete in each period, and it can automatically save and securely encrypt and send the training data to the research server *via* an internet connection.

#### Attentional Control Training Program Sham Training *via* a Smartphone Application

The AACTP sham control training is like the AACTP in that it is delivered *via* a smartphone application based on an Android or iOS application and is designed in accordance with the conventional program. Patients receiving the sham control program will also have the application installed on their personal smartphone. They, therefore, will be able to use the program in any location that they wish. However, different from the AACTP, patients in the active control group will be trained to direct their attention toward the non-alcohol-related stimuli on only 50% of the trials. On the remaining trials, their attention will be directed toward the alcohol-related stimuli. Thus, the sham training should counteract the induction of AB with AB re-training because the allocation of the participant's attention will distribute equally between the alcohol-related and the non-alcohol-related stimuli. The patient will respond by touching the circle displayed on the computer screen as quickly and accurately as possible. Like the ACTP training, a red outline will signal a stimulus that should be ignored, and a green outline will signal a neutral stimulus, which should be responded to, but the alcoholic or non-alcoholic stimuli will appear equally often with the red or green outline. Also, throughout each session, the speed at which the stimuli are presented will systematically increase, and the width of the outlines will decrease and gradually fade away in the final sessions, thereby increasing the difficulty of the task. In case the patient makes an error and touches an incorrect stimulus, the tablet will provide an auditory-visual alert. As in the smartphone version of the alcohol ACTP, prior to each session the participant will be encouraged to set a goal for the speed of the reaction times and the number of errors made until his or her performance plateau has been reached. After completing each session, the patient will be given both graphical and auditory feedback. Hence, the program will contain all the same features as the smartphone version of the alcohol ACTP. The only difference is that the patient's attention will be equally guided toward non-alcohol-related and alcohol-related stimuli in the sham control version. Selected practice data will be sent to the server to control for dose-response effects. Having the sham condition is important for ensuring that effects obtained with the smartphone version of the alcohol ACTP result from the re-training of the patients' alcohol attentional bias and not from the additional attention that they receive or from some other unintended effect, such as the Hawthorne effect (53).

#### Treatment as Usual

Patients in the Treatment-As-Usual (TAU) (see Section 2.2.) control group will not receive any kind of additional treatment, because we aim to determine whether the smartphone version of the alcohol ACTP will increase the effectiveness of a well-established, evidence-based pharmacological and psychological treatment.

### Measures

Upon admission to the study, patients in all three or the groups will be assessed in a baseline interview. Details about patients' socio-demographic characteristics, AUD diagnosis, treatment goals, and pharmacological treatment will be obtained from their clinical records (54–56). At baseline, the general premorbid intelligence level and cognitive functioning will be assessed. Experimental measures of patients' cue-induced cravings, alcohol AB, action-tendency bias, inhibition bias, and cognitive flexibility (24, 26, 27, 57, 58) will be administered at baseline and post-treatment. Clinical measures of patients' alcohol consumption (the primary outcome measure), cravings, self-efficacy, and affective states (the secondary outcome measures) (57–62) will be taken at the baseline, post-treatment, and follow-up assessments. The follow-ups will be given at 3- and 6-months post-treatment.

#### Baseline Measures of General Cognitive Functioning

*National Adult Reading Task*. The Danish version of the National Adult Reading Task (DART/NART) (63) assesses verbal crystalized intelligence, which is indicative of general premorbid cognitive functioning. Respondents are asked to read 50 incongruent words in Danish (i.e., the pronunciation does not correspond with how the word is spelled). The total DART score can be converted to an intelligence quotient score (IQ score).

*Montreal Cognitive Assessment*. The Montreal Cognitive Assessment (MOCA) is a short screening tool that is sensitive to mild cognitive impairment. It assesses six cognitive domains: attention, orientation, executive functioning, visuospatial construction, memory, and language (64). The MOCA consists of short cognitive tasks that can be administered in 10 min.

#### Experimental Measures

##### Cue-Induced Cravings

Cravings for alcohol will be assessed by exposing patients to 14 alcohol-related pictures from the AACTP and 14 alcohol-related pictures that were not included in the AACTP. The aim is to determine whether cue-induced cravings will decrease in the experimental AACTP group and whether the decrease is generalized to alcohol-related stimuli not included in the AACTP. Cue-induced cravings in response to the pictures will be measured using (a) self-report by means of a visual-analog scale (see Section 2.6.2.2. for a description) (57, 58) and (b) physiological reactivity by means of skin conductance response using Imotions, which is a computerized system that has been used in various experimental studies.

##### Cognitive Flexibility

The classic Stroop task will be used to measure patients' degree of general attentional control. Task stimuli consisting of two types of words are used: (1) Congruent words comprise the names of four colors (i.e., red, yellow, blue, and green) that are written in a color that is congruent with the name of the color (e.g., the word *red* in red letters); (2) Incongruent words consist of the same four names of colors, but they are presented in a font color that is incongruent with the name of the color (e.g., the word *red* in blue letters). The patient's task is to name the color in which the words are presented while attempting to ignore the meaning of the word. Both the mean reaction time for correctly naming the color of the words and the number of correct responses (mean accuracy) will be recorded. Subtracting mean reaction time to the congruent words from the mean reaction time to the incongruent words gives a measure of the participant's cognitive interference (26).

##### Alcohol Attentional Bias

Patients' AB for alcohol cues will be assessed using the alcohol Stroop task. The task consists of two categories of words presented in different font colors (i.e., red, yellow, blue, and green): (1) alcohol-related words (e.g., bar, beer, rum, scotch, tequila, vodka, whisky), and (2) neutral words (e.g., ceiling, cupboard, fence, gate, shed, tap, chair). The two categories of words are matched for word frequency and length, number of syllables, and semantic relatedness. As in the classic Stroop task, the patient's task will be to name the color in which the words are presented while attempting to ignore the meaning of the words. AB is indicated by longer reaction times to the alcohol-related words relative to the neutral words (27, 32).

In this study, the alcohol Stroop task with pictures will also be used to measure patients' AB for alcohol cues, thereby indicating whether patients have benefitted from the AACTP training. To determine this, the task will consist of pictures of alcoholic and non-alcoholic beverages, each presented with colored outlines (i.e., red, yellow, blue, or green): (1) pictures of alcoholic beverages (e.g., beer, wine, spirits), and (2) pictures of non-alcoholic beverages (e.g., water, orange juice, smoothie). Alcohol AB is indicated by longer reaction times to the alcohol cues than to the non-alcoholic cues (24).

##### Attentional Bias Measured With Eye Tracker

*Multi-stimulus free-viewing task (MSFVT)*. The MSFVT will be used to also measure patients' AB for alcohol cues. The task consists of 54 matrices, each containing pictures of eight alcoholic and eight non-alcoholic drinks. Each matrix will be presented for 6 s. The task consists of three blocks of 18 trials. Unlike the alcohol Stroop task, this task is not based on reaction times; instead, it involves eye tracking. For each matrix, the patient is required to gaze at a fixation dot in the center of the screen for 100 ms for the matrix to appear. After a further 2,000 ms interval, the next fixation dot appears (65, 66). Tracking of the patient's eyes will, like the other physiological measure (skin conductance response), be recorded using Imotions.

##### Alcohol Action Tendency and Inhibition Bias

*Alcohol approach-avoidance task (AAT)*: The AAT measures the degree of alcohol approach bias. During the AAT, patients are requested to react to pictures of the alcoholic drinks by using avoidance responses (i.e., by pushing a joystick away from themselves) and to react to the pictures of non-alcoholic drinks using approach responses (i.e., by pulling a joystick toward themselves). The mean reaction time and the number of correct approach and avoidance responses will be recorded. Similar to AB, approach bias is indicated when reaction times are faster for approaching alcohol cues than for avoiding them, whereas the opposite indicates an avoidance bias (67, 68).

Alcohol Go/No-Go (A-GNG) task: Patients' response inhibition for alcohol cues will be recorded with the A-GNG (69, 70). Here, the patient must respond to pictures of non-alcoholic drinks (e.g., a bottle of coke or of water; the Go stimuli) and inhibit their responses to alcoholic stimuli (e.g., a bottle of beer; the No-Go stimuli). The following indices will be recorded: Number of commission errors (i.e., *false alarms* or erroneously responding to no-go stimuli), omission errors (i.e., *misses* or failing to react on go-stimuli), accuracy (i.e., *hits* or the number of correct responses for go-stimuli), and the reaction times for the trials with correct response on go-trials. A high rate of commission errors would indicate that the patient has reduced inhibitory control, whereas a large number of omission errors would indicate lapses in attention (71). To avoid potential practice effects from the baseline to the post-treatment assessments of the experimental tasks, two parallel versions of the experimental tasks will be used.

#### Clinical Outcomes

##### Primary Outcome Measures

Alcohol consumption will be measured with the Alcohol Timeline Follow-back (TLFB) method. It involves using a calendar to help the patient retrospectively recall the number of drinks that he/she consumed on each day during the previous 3 months (59, 72). The results will be used to calculate various alcohol consumption measures, including weekly mean drinking, which will be the primary outcome measure.

To validate the TLFB, hair samples from the patients will be tested for ethyl glucuronide (ETG) by liquid chromatography-tandem mass spectrometry (LC-MS/MS). This biological marker of alcohol consumption will be collected and analyzed according to the Society for Hair Testing (73, 74).

##### Secondary Outcome Measures

A visual analog scale (VAS) will be used to measure patients' alcohol cravings on a scale ranging from *0* to *10*, with *0* indicating no craving at all and *10* indicating extreme craving. The scale will be presented visually on a ruler, and patients will be asked to indicate their mean and peak level of craving during the past 30 days (57, 58).

The Alcohol Abstinence Self-Efficacy Scale is a 40-item measure of patients' temptation to drink and their perceived self-efficacy in abstaining from drinking in 20 different situations that represent typical cues for drinking. Twenty items pertain to temptation, and 20 items pertain to self-efficacy. Patients will rate each item on a scale ranging from *not at all* (0) to *extremely* (4). The measure comprises the following sub-scales: (1) negative affect; (2) social interaction and positive states; (3) physical and other concerns; and (4) withdrawal and urges to drink. Both the temptation and perceived efficacy total score can range from 0 to 80 (60).

The Readiness-to-Change Questionnaire Treatment Version (RTCQ-TV) is a 12-item measure of patients' stated intentions to change their drinking, which includes the following sub-scales: (1) pre-contemplation, (2) contemplation, and (3) action stages. Four items pertain to each sub-scale, and each item is rated on a 5-point Likert Scale ranging from strongly agree (−2) to strongly disagree (+2). The total score can range from −24 to +24 (61, 75).

The Positive and Negative Affect Schedule (PANAS) is a 20-item measure of the patient's affective states, which includes two sub-scales: (1) positive affect and (2) negative affect. Ten items pertain to each sub-scale, and each item is rated on a 5-point scale ranging from *1* (*not at all*) to *5* (*very much*). The total score can range from 20 to 100 (62).

### Statistical Analysis

The intent-to-treat principle will be followed in the analyses to evaluate the intervention. A repeated-measures MANOVA with groups as the between-participants factor and assessment time-point as the within-participants factor will be used to test the efficacy of the intervention across the assessment points. Efficacy would be indicated by a significant Time X Study Group interaction. Each of the significant interactions will be followed up with *post-hoc* tests to identify the source of the interaction. Potential demographic covariates (e.g., age, education, income) will also be considered by using MANCOVA. Further, attrition rate at each follow-up time point will be evaluated. Baseline characteristics of participants who remain in the study will be compared with those who have been lost to follow-up. A multiple imputation (MI) approach that adjusts for uncertainty arising from missing data (76, 77) will also be conducted to evaluate the main findings.

Based on MacKinnon (78) mediational procedures, a series of regression analyses will be conducted to test the hypothesized mediational relationships among the outcome variables, i.e., that reductions in *alcohol AB* and *cravings* will partially mediate improvements in the outcome measures. Several ancillary interaction effects will also be evaluated, although they are not hypothesized to be significant. They include potential interactions between the intervention received and patients' drinking history, affective states, and cognitive flexibility. Although the intervention is intended to be effective for all AUD patients, it is important to test this assumption by evaluating whether the intervention is effective for specific sub-groups of patients.

A power analysis (79, 80) was conducted using G^*^Power for a repeated-measures MANOVA with α = 0.05; effect size (ES) of *f* =0.20; power = 0.90; groups *k* = 3; and time-points measurements = 4. It showed that 88 participants will be required for the final analyses in each of the four cells (total *N* = 264). Prior evaluations identified *f* s of 0.30 (AB) and 0.37 (weekly mean drinking) and an *f* of 0.42 (situational confidence to resist drinking) on outcome measures for sub-clinical excessive drinkers (36). Further, in a study that used the intervention as an add-on treatment for clinical participants with a substance-use disorders other than AUD, effect sizes of *f* s = 0.33 (drug-specific AB), 0.34 (number of relapses), 0.32 (temptation to use) and 0.37 (self-confidence to resist temptation) were identified (42). Also, an unpublished study conducted with detoxified drug-abusers found *f* s of 0.39 (drug-specific AB), 0.18 (number of relapses), and 0.35 (temptation to use) (81). A second power analysis for a MANOVA with special effects and interactions was conducted to test the dosage-effect across groups (α = 0.05; EF *f* ^2^(*v*) = 0.06; power = 0.90; *k* = 3; predictors = 3; and response variables = 8), which yielded an *N* of 220. Attrition in similar studies has usually ranged from 10 to 15% (41, 42). Assuming a somewhat higher attrition rate (20%), which is not unlikely (82, 83), indicates that 317 (264 + [264·0.20]) participants should be tested at baseline for an analysis using listwise deletion of missing cases, although a multiple imputation (MI) analysis will also be conducted to verify that results converge across missing data assumptions. The projected sample size is sufficient for the planned data analyses, including mediational analyses based on Fitz and MacKinnon's guidelines (84).

### Ethics

The patients in this study will undergo primary treatment in the outpatient clinics. Although 66% of them will not receive additional treatment, all patients will be treated with the standard evidence-based AUD treatment. Thus, we find no ethical problems with not offering the add-on intervention to the entire sample. However, a critical ethical question is whether use of the ACTP would encourage patients randomized to the experimental group to consume alcohol instead of discouraging them for doing so. This concern relates to the fact that ACTP involves *in vivo* exposure to alcohol through the alcohol-related pictures. However, in Western cultures, all patients will be continuously exposed to alcohol cues, both during and after treatment, and they will be unable to avoid this because large-scale alcohol advertisements are continuously on public display in magazines and on television. Also, in Denmark alcohol is readily available, highly visible, and easy to buy around the clock in supermarkets, delicatessens, kiosks, and service stations. We, therefore, consider exposure to the alcohol pictures in the experimental group to be no riskier for patients than exposure to them in everyday life. By contrast, in the experimental group, the patients will be trained to focus less on alcohol cues and more on non-alcoholic drinks than in real life. Furthermore, the ACTP will be available only during the opening hours of the alcohol outpatient clinics, and patients in the experimental groups will be provided with a direct telephone number of a therapist in case they experience uncontrollable cravings. Patients in all three of the groups will have the option to call a therapist. All attempts will be taken to intervene in case a patient relapse. The protocol has been approved by the Regional Scientific Ethical Committees for Southern Denmark (Project ID: S-20200200).

## Discussion

The present study will investigate whether (1) the AACTP as an add-on intervention increases the effectiveness of a primary evidence-based treatment, and (2) a gamified version of AACTP can be successfully delivered *via* a smartphone application as an add-on. As mentioned in the introduction, the alcohol ACTP has been evaluated in community samples of excessive drinkers, for whom reductions in AB and alcohol consumption were observed post-treatment and at follow-up (32, 36, 37). In the first experimental study, it was found that individuals with harmful alcohol use (drinking > 50 units per week for men and > 35 units per week for women) showed reductions in both AB and alcohol consumption at post-treatment, and these reductions were maintained at a 3-month follow-up (32). In this initial study, the participants served as their own controls, which is a commonly used method in an initial evaluation of a new intervention (85). Hence, after recruitment and assessment, there was, in accordance with this alternative tradition, a waiting period before ACTP was started, and during this period there was no change in either AB or alcohol consumption, but there was a decline in both measures following the introduction of the AACTP intervention 1 month later (32). Similarly, a second randomized controlled study of a sub-clinical sample of hazardous drinkers (22–50 units per week for men and 15–35 units per week for women) and persons with harmful alcohol use found that the ACTP (compared to a non-active control group) was effective in decreasing alcohol consumption at post-treatment and at the 3-month follow-up, but the reductions had attenuated at the 6-month follow-up (36). A third randomized controlled study investigated whether the AATCP could be successfully delivered *via* a fully automated web-based delivery pathway to a community sample of hazardous drinkers. At post-intervention, a reduction in both alcohol craving and alcohol consumption was found in the AACTP group compared to an active sham-control group. However, the effects on alcohol consumption were not maintained at the 3-month follow-up (37). Finally, a fourth study tested a gamified AACTP delivered though a smartphone application in another community sample of hazardous drinkers (18–40 years old). Results indicated that reductions of up to 40% in alcohol consumption had occurred several months after the training (86).

The fact that the AACTP has to date been tested only in community samples of excessive drinkers highlights the need for this program to be tested in clinical AUD samples to determine its effectiveness as a clinical treatment. There are various facets of the training that might need to be altered when using it in a clinical setting. For instance, it might be necessary to increase the number of the AACTP training sessions, because clinical samples have a stronger AB than community samples, and the AACTP might have specific dosage effects. Furthermore, whereas prior studies conducted with community samples have treated individuals only with the AACTP, this may not be sufficient for treating AUD patients in a clinical setting. For instance, it might be that the AACTP works best when it is used as an add-on to an existing treatment. That is, changing the attentional pattern of selective attention for alcohol cues may be essential but not sufficient for bringing about enduring changes in the addictive behavior. Combining the AACTP with another treatment, such as CBT, might be more effective in the long term than would treatment when delivered separately. Combining psychological treatments that address AUD-related cognitive dysfunctions in the *reflective* and *impulsive* systems might better prepare patients for their inevitable confrontation with alcohol cues in their natural environment and increase the probability of preventing relapses in the longer term. In short, rigorous, and well-powered clinical studies are needed before firm conclusions can be reached about whether the AACTP is an effective clinical treatment.

In recent years, advances on the internet and mobile technologies have made it possible for neuropsychological treatments, such as the AACTP, to be used outside the laboratory, and several pioneering studies have been conducted to test the effects of this approach. For instance, as mentioned earlier, Wiers et al. (37) administered the AACTP over the Internet in its conventional form and found a reduction in users' alcohol consumption (37). Cox et al. (36) administered a gamified AACTP smartphone application and also found both short-term and longer-term reductions in alcohol consumption (86). The AACTP in its original form has some gamification elements (e.g., a progression in level of difficulty, goal-setting, feedback, and time pressure), and Wiers et al.'s (37) study along with other pioneering studies highlight the potential for cognitive-bias modification to be delivered over the Internet.

Clinical neuropsychological treatments, including the AACTP, have shown great promise in treating cognitive dysfunctions not targeted by conventional psychological treatments, and the use of gamification elements may also enhance compliance and the effectiveness of these interventions, as Cox et al. (36) initially confirmed with their gamified version of the AACTP. However, as these authors also note, it is important to be mindful of the target audience when delivering a gamified neuropsychological treatment.

The use of the Internet and mobile devices to deliver a novel treatment to adults might help us improve standard evidence-based treatments for AUD and related disorders (2–4). However, although eHealth-based interventions might have major advantages in terms of increasing availability and reducing the socio-economic burden on society, when targeting clinical AUD populations, these interventions should be delivered only as an add-on for increasing the effectiveness of a treatment that has already been proven to be effective. Because AB for alcohol cues is proportional to the amount of alcohol habitually consumed, and these biases are not addressed within current evidence-based treatment programs, this study is expected to provide new evidence regarding the effectiveness of the gamified AACTP in a clinical population. Furthermore, the intervention should also provide valuable insights into implicit cognitive processes and relationships among alcohol ABs, action-tendency bias (i.e., approach-avoidance bias), and inhibition bias. These implicit processes have all been studied extensively in patients with AUD (16, 25, 87), but there is still a need for more integrative evidence on the associations between the various types of alcohol-related cognitive biases and their role in the maintenance of addictive behaviors (88).

If this study demonstrates that AACTP is effective in patients with AUD, other means of assessing ABs and delivering cognitive training programs could be implemented by utilizing technologies such as virtual reality (VR) and augmented reality [AR; (89)]. These technologies have already been developed for targeting approach biases through the approach avoidance training program (90). Paradigms using VR and AR might overcome some of the shortcomings of the AACTP by increasing its ecological validity. The multimodal nature of these paradigms might cause them to be perceived as closer to real-life situations than other eHealth options (91). Nonetheless, the implementation of VR and AR technologies is an emerging field of research, and the exact implications of their efficacy and effectiveness in treatments targeting AB in AUDs are yet to be examined (91).

Overall, the promising results from the use of AACTP in community samples suggest that the AACTP treatment in this study will be effective, thus paving the way for such a treatment to be implemented in clinical settings. We expect that this study will increase the effectiveness of an existing evidence-based AUD treatment (i.e., CBT) and that it will show that a low-cost and easily available gamified treatment is also effective. Thus, this study is likely to have an impact at the scientific, clinical, and societal level.

## Ethics Statement

The protocol has been approved by the Regional Scientific Ethical Committees for Southern Denmark (Project ID: S-20200200). All participants will be provided with oral and written information about the study and sign written informed consent before enrollment in the study.

## Author Contributions

All authors contributed to the conception and design of the study.

## Funding

The study was funded by the Trygfonden.

## Conflict of Interest

The authors declare that the research was conducted in the absence of any commercial or financial relationships that could be construed as a potential conflict of interest.

## Publisher's Note

All claims expressed in this article are solely those of the authors and do not necessarily represent those of their affiliated organizations, or those of the publisher, the editors and the reviewers. Any product that may be evaluated in this article, or claim that may be made by its manufacturer, is not guaranteed or endorsed by the publisher.

## Abbreviations

AASE: alcohol abstinence self-efficacy scale
AAT: Alcohol approach-avoidance task
AB: attentional bias
A-GNG: Alcohol Go/No-Go
ACTP: attention control training program
AUD: alcohol use disorder
CBT: cognitive behavior therapy
ETG: ethyl glucuronide
LC-MS/MS: liquid chromatography-tandem mass spectrometry
MSFVT: Multi-stimulus free-viewing task
MoCA: Montreal Cognitive Assessment.
PANAS: Positive and Negative Affect Schedule
RTCQ-TV: Readiness-to-Change Questionnaire Treatment Version
RT: reaction time
TAU: treatment as usual
TLFB: timeline follow-back
VAS: Visual Analog Scale.
